# Risk Factors for Acquired *Stenotrophomonas maltophilia* Pneumonia in Intensive Care Unit: A Systematic Review and Meta-Analysis

**DOI:** 10.3389/fmed.2021.808391

**Published:** 2022-01-12

**Authors:** Neng Wang, Congchen Tang, Lichun Wang

**Affiliations:** Center of Infectious Disease, West China Hospital, Sichuan University, Chengdu, China

**Keywords:** *Stenotrophomonas maltophilia*, ICU-acquired pneumonia, risk factor, meta-analysis, infection

## Abstract

**Background and Aims:** *Stenotrophomonas maltophilia* is increasingly found in critically ill patients, but it is considered a pathogen of limited pathogenicity and therefore it is not often targeted. We systematically evaluated risk factors for *S. maltophilia* pneumonia in ICU patients for better clinical management.

**Methods:** Prospective and retrospective studies of *S. maltophilia* infection in the ICU from database establishment to August 8, 2021, were searched through PubMed, web of science, Cochrane Library Embase and CNKI. The literature was independently screened and extracted by two authors according to inclusion and exclusion criteria, evaluated for quality by the NOS scale, and meta-analyzed by stata 14.0 software.

**Results:** A total of eight studies with a sample size of 2,320 cases were included. Meta-analysis showed that APACHE-II score > 20 (OR = 10.98, 95% CI: 5.67 ~ 21.26), COPD (OR = 3.97, 95% CI: 2.39 ~ 6.61), malignant tumor (OR = 2.15, 95% CI: 1.03 ~ 4.50), mechanical ventilation (OR = 8.75, 95% CI: 2.59 ~ 29.58), tracheotomy (OR = 6.12, 95% CI: 2.06 ~ 18.18), endotracheal intubation (OR = 4.25, 95% CI: 2.30 ~ 7.84), β- Lactamase inhibitors (OR = 9.98, 95% CI: 1.51 ~ 65.96), aminoglycosides (OR = 4.01, 95% CI: 2.06 ~ 7.80), carbapenems (OR = 2.82, 95% CI: 1.49 ~ 5.31), and quinolones (OR = 2.17, 95% CI: 1.21 ~ 3.89) were risk factors for ICU-acquired *S. maltophilia* pneumonia.

**Conclusion:** Many risk factors are associated with *S. maltophilia* pneumonia in ICU patients. Clinical workers should pay more attention to assessing the risk of infection in ICU patients and enhance the prevention and management of high-risk groups, which will help reduce their risk of *S. maltophilia* infection.

## Introduction

*S. maltophilia* is a non-fermentable Gram-negative bacterium that is an opportunistic agent. It is naturally resistant to many commonly used antibiotics, such as carbapenems and aminoglycosides (1, 2). It is due to such characteristics, in the context of drug resistance, that *S. maltophilia* is becoming an important pathogen of hospital infections in the ICU, which can lead to infections in the lungs, bloodstream and many other important parts of the body, even life-threatening (3, 4).

According to the CHINET bacterial resistance surveillance data in 2020, *S. maltophilia* accounted for 2.98% of all strains and ranked 9th, 7th among Gram-negative bacteria, and 3rd among non-fermentative bacteria, after *Bacillus immobilis* and *Pseudomonas aeruginosa*. *S. maltophilia* has the highest resistance to ceftazidime (38.5%), followed by levofloxacin (10.8%), compound sulfamethoxazole (6.7%) and tigecycline (2.7%), and the lowest resistance to minocycline (2.3%) (5). Clinically, it often causes mixed infections with other bacteria, mainly *Pseudomonas aeruginosa, Klebsiella pneumoniae*, and *Acinetobacter baumannii*, so *S. maltophilia* infections are difficult to be treated and the mortality rate is high. Muder et al. (6) reported a mortality rate of 21% in patients with *S. maltophilia* bacteremia. Paez et al. (7) reported a direct mortality rate of 26.7% due to *S. maltophilia* infection and a mortality rate of 21–69% associated with *S. maltophilia* infection.

The study of risk factors for ICU-acquired *S. maltophilia* pneumonia is of great practical significance for the in-depth study of the hazards of this bacterium and the adoption of appropriate preventive and control measures. Currently, Several studies at home and abroad have investigated the risk factors for the occurrence of *S. maltophilia* infection in ICU patients, but there are drawbacks such as small sample size and incomplete risk factor indicators, and the significance of clinical guidance is limited. This study aims to systematically evaluate the risk factors of hospital-acquired *S. maltophilia* pneumonia by Meta-analysis, and provide a theoretical basis for clinical formulation of prevention and control strategies to reduce the morbidity and mortality of *S. maltophilia* infection.

## Materials and Methods

### Literature Search Strategy

PubMed, Web of Science, Cochrane Library, Embase and CNKI were searched from the time of database establishment to August 8, 2021. A combination of subject terms, free words, and Boolean logical operators was used for the search terms: *Stenotrophomonas maltophilia*, hospital-acquired pneumonia, ventilator-associated pneumonia, intensive care, risk factors, etc. The English databases were searched for the following terms: (*Stenotrophomonas maltophilia* OR *S. maltophilia* OR SMA) AND (nosocomial infection OR hospital infection OR hospital acquired infection OR cross infection OR VAP OR ventilator associated pneumonia OR ventilator-associated pneumonia) AND (ICU OR Intensive Care OR NICU OR PICU OR CCU) AND (risk factor OR factor). A manual search of relevant content reviews and references of included literature was conducted to identify potential studies that met the inclusion criteria.

### Literature Inclusion and Exclusion Criteria

The inclusion criteria were as follows: (1) The type of literature was a cohort study or case-control study published nationally and internationally; (2) The study population was divided into two groups based on whether they were infected with *S. maltophilia*, and the diagnosis criteria for *S. maltophilia* pneumonia in this study were described below; (3) Risk factors for *S. maltophilia* pneumonia in ICU patients were present in the literature, such as comorbid underlying diseases, invasive procedures undergone, and use of broad-spectrum antibiotics; (4) Outcome indicators for risk factors for *S. maltophilia* pneumonia in ICU patients could be expressed as odds ratios (OR), and their 95% confidence intervals (CI) were calculated. Studies were excluded when meeting one of the following criteria: (1) duplicate reports, conference reports, and reviews; (2) abnormal or missing data; (3) low quality of literature [Newcastle-Ottawa Scale (NOS) score ≤ 3].

In this study, infection and colonization were considered ICU-acquired if they were diagnosed more than 48 h after ICU admission. Pneumonia was defined as follows: (1) new or progressive pulmonary infiltrates. (2) Temperature > 38°C or <36.5°C, leukocyte count >12,000 μl^−1^ or <4000 μl^−1^, purulent endotracheal aspirate or sputum. (3) Positive respiratory sample. (4) Decreased oxygenation.

### Data Extraction and Quality Evaluation

The retrieved literature was screened by two authors independently according to the inclusion and exclusion criteria, and the following data information was extracted: name of the first author, time of publication, source of the literature, basic characteristics of the included cases, and possible risk factors for *S. maltophilia* pneumonia in ICU patients. If the opinions of two reviewing authors do not agree, they discuss. If there was still disagreement after discussion, a third party opinion was sought. The quality of the included literature was also evaluated according to NOS score (8), and the evaluation items include three aspects of population selection, comparability and exposure evaluation, with a score out of 9. A score of 7 and above was considered as high-quality literature, 4–6 as moderate quality literature, and 1–3 as low-quality literature.

### Data Analysis

Statistical analysis was performed by stata14.0 software. *I*^2^ was used to determine the heterogeneity of the included literature, and the fixed-effects model was used when p > 0.1 and *I*^2^ < 50%; otherwise, the random-effects model was applied. OR and its 95% CI were calculated for count data, while weighted mean difference (WMD) and its 95% CI were calculated for measurement data, and differences were considered statistically significant at *p* ≤ 0.05. Sensitivity analysis was performed by calculating the OR and 95% CI for both fixed-effects and random-effects models and comparing the results of the two groups. Sensitivity analysis was performed by changing the data analysis model. If there was no substantial change after the model change (no opposite conclusion was reached after changing the model), the consolidated result was considered to be stable. Begg′s test was used to test for publication bias when the number of included papers for individual risk factor analysis was ≥3.

## Results

### Literature Search

**Figure 1:**
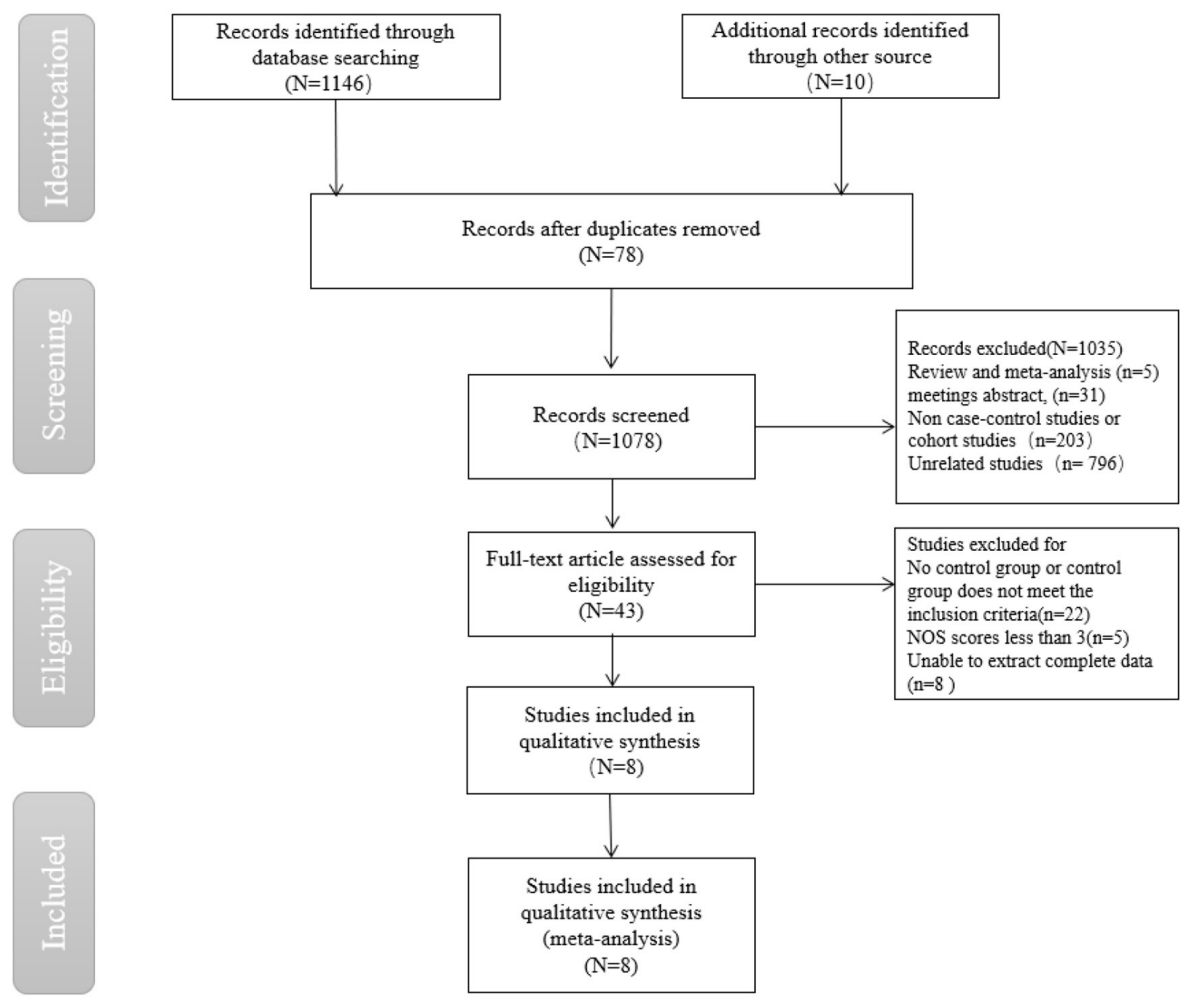
Flow chart of literature screening for meta-analysis on risk factors for acquired *Stenotrophomonas maltophilia* in intensive care unit.

Firstly, 1,156 papers were initially searched in the database through the search strategy, and then 43 papers were selected through title, abstract and keywords, etc. Finally, eight papers (9–16) were further screened by reading the full text, including five papers in English and three papers in Chinese (Figure 1).

### Baseline Characteristics of the Studies

The eight included papers included in the study were published between 2002 and 2020, seven of which were case-control studies and one was a cohort study involving 2320 patients, 306 in the *S. maltophilia*-infected group and 2014 in the non-infected group, and 25 exposure factors for *S. maltophilia* infection were extracted. The quality of the eight papers was evaluated using the NOS scale, including five high-quality papers and tree moderate-quality papers. The basic characteristics of the included literature are shown in Tables 1, 2.

**Table 1:** Characteristics of the included studies.

**Study**	**Year**	**Design study**	**Area**	**Infection group**	**Non-infection group**	**Risk factors**
Stang et al. (8)	2002	Case-control	USA	26	137	01.02.03.07.20.21.22.24
Hanes et al. (9)	2006	Cohort	France	30	60	01.02.07.08.09.11.14.15.16.17.18.21.22.23.24.25
Nseir et al. (10)	2011	Case-control	China	35	140	01.02.04.11.14.15.16.19.21.23
Xu et al. (11)	2012	Case-control	Germany	36	28	01.02.03.08.09.10.12.13.14.15.17.18
Guo et al. (12)	2014	Case-control	China	42	84	01.02.04.06.08.09.11.14.15.16.19
Saugel et al. (13)	2016	Case-control	Netherlands	6	15	01.02.03.08.09.10.11.13.14.16
Ibn Saied et al. (14)	2019	Case-control	China	29	58	01.02.03.09.10.11.13.14.17.18.21.22.23.24.25
Lei et al. (15)	2020	Case-control	USA	102	1,492	01.02.05.06.07.11.12.13.20

*01, Age, years; 02, Gender; 03, APACHE-II score; 04, APACHE-II score >20; 05, Glasgow score; 06, Glucocorticoid; 07, Length of ICU stay, days; 08, COPD; 09, Diabetes; 10, Malignancy; 11, Cardiovascular disease; 12, kidney dysfunction; 13, Immunosuppression; 14, Mechanical ventilation; 15, Tracheal intubation; 16, Tracheotomy; 17, Central venous catheterization; 18, Urinary catheter; 19, Nasogastric tube; 20, Operation; 21, Carbapenems; 22, β-lactamase inhibitor; 23, Aminoglycosides; 24, Quinolones; 25, Nitroimidazoles*.

**Table 2:** Risk of bias assessment of the included studies according to the Newcastle-Ottawa Scale (NOS).

**NOS items/Study ID**	**Hanes et al**.	**Xu et al**.	**Saugel et al**.	**Guo et al**.	**Scholte et al**.	**Shi et al**.	**Saied et al**.
Is the case definition adequate?	^*^		^*^	^*^	^*^	^*^	^*^
Representativeness of the cases	^*^	^*^	^*^	^*^	^*^		^*^
Selection of controls	^*^	^*^	^*^		^*^	^*^	^*^
Definition of controls	^*^	^*^	^*^	^*^	^*^	^*^	^*^
Compatibility	^*^	^*^	^*^	^*^	^*^	^*^	^*^
Ascertainment of exposure	^*^	^*^	^*^	^*^	^*^	^*^	^*^
Same method of ascertainment for cases and control	^*^	^*^	^*^	^*^			^*^
Non-response rate	^*^	^*^			^*^	^*^	^*^
Total score	8	7	7	6	7	6	8

* *Representative studies meet this criteria*.

### Meta-Analysis of Exposure Factors for *S. maltophilia* Pneumonia

Heterogeneity was tested for exposure factors such as age, gender, APACHE-II score, length of ICU stay, Glasgow score, glucocorticoid use, diabetes, malignancy, cardiovascular disease, renal insufficiency, immunodeficiency disorders, tracheal intubation, indwelling catheters, surgery, and use of carbapenems, quinolones, and aminoglycosides. Heterogeneity was acceptable (*p* > 0.10, *I*^2^ < 50%), and effect sizes were combined using a fixed-effects model. heterogeneity was present for APACHE-II scores >20, COPD, tracheotomy, mechanical ventilation, indwelling nasogastric tube, central venous line, use of β-lactamase inhibitors and nitroimidazole antibiotics (*p* < 0.10, *I*^2^ > 50%), random-effects model combinations of effect sizes were performed.

The meta-analysis showed that risk factors for *S. maltophilia* pneumonia in the ICU included APACHE-II score > 20 (OR = 10.98, 95% CI: 5.67 ~ 21.26), COPD (OR = 3.97, 95% CI: 2.39 ~ 6.61), malignant tumor (OR = 2.15, 95% CI: 1.03 ~ 4.50), mechanical ventilation (OR = 8.75, 95% CI: 2.59 ~ 29.58), tracheotomy (OR = 6.12, 95% CI: 2.06 ~ 18.18), endotracheal intubation (OR = 4.25, 95% CI: 2.30 ~ 7.84), β- Lactamase inhibitors (OR = 9.98, 95% CI: 1.51 ~ 65.96), aminoglycosides (OR = 4.01, 95% CI: 2.06 ~ 7.80), carbapenems (OR = 2.82, 95% CI: 1.49 ~ 5.31), and quinolones (OR = 2.17, 95% CI: 1.21 ~ 3.89). There was no obvious correlation between risk factors and *S. maltophilia* pneumonia, such as age, gender, APACHE-II score, length of stay in ICU, combined diabetes, combined cardiovascular disease, combined renal insufficiency, combined immunodeficiency disease, indwelling catheter, operation, central venous line, indwelling nasogastric tube, corticosteroids, and nitroimidazole antibiotics.

### Sensitivity Analysis and Publication Bias

Sensitivity analysis suggested that the results of the meta-analysis were stable for all outcome indicators except for two exposure factors, central venous placement and indwelling nasal cannula (Table 3). Begg's test was used to test for publication bias when the number of included papers for individual risk factor analysis was ≥3. The results showed *p* > 0.05, indicating that the publication bias of included papers was not significant.

**Table 3:** Meta-analysis results of risk factors for acquired *Stenotrophomonas maltophilia* in intensive care unit.

**Exposure factors**	**Included studies**	**Heterogeneity**	***p***	**Fixed-effect model (FEM)**	***p***	**Random-effect model (REM)**	***p***
**General condition**							
Age, years	8	0	0.93	−0.76 (−2.62~1.10)	0.42	−0.76 (−2.62~1.10)	0.42
Gender	8	0	0.83	0.77 (0.59~1.02)	0.07	0.77 (0.58~1.01)	0.06
APACHE-II score	3	0	0.83	2.80 (−0.31~5.82)	0.08	2.80 (−0.31~5.82)	0.08
APACHE-II score >20	2	0	0.39	10.98 (5.67~21.26)	<0.001	11.49 (6.02~21.92)	<0.001
Glasgow score	2	0	0.66	−0.50 (−1.91~0.90)	0.49	−0.50 (−1.91~0.90)	0.49
Glucocorticoid	3	42	0.58	0.91 (0.51~1.61)	0.74	0.98 (0.42~2.29)	0.97
Length of ICU stay, days	4	0	0.50	1.65 (0.70~2.60)	0.001	1.65 (0.70~2.60)	0.001
**Pre-existing medical conditions**							
COPD	4	77.8	0.004	3.97 (2.39~6.61)	<0.001	3.99 (1.19~13.32)	0.03
Diabetes	5	8.1	0.36	1.50 (0.89~2.63)	0.13	1.47 (0.82~2.61)	0.20
Malignancy	3	0	0.99	2.15 (1.03~4.50)	0.04	2.15 (1.03~4.50)	0.04
Cardiovascular disease	7	48.2	0.07	0.92 (0.66~1.29)	0.63	1.0 (0.61~1.75)	0.92
kidney dysfunction	3	0	0.87	1.20 (0.69~2.07)	0.52	1.21 (0.70~2.07)	0.50
Immunosuppression	3	49.6	0.14	1.38 (0.87~2.21)	0.17	1.70 (0.38~7.69)	0.49
**Invasive procedures**							
Mechanical ventilation	5	71.4	0.007	8.22 (4.82~14.03)	<0.001	8.75 (2.59~29.58)	<0.001
Tracheal intubation	3	0	0.52	4.25 (2.30~7.84)	<0.001	4.08 (2.22~7.51)	<0.001
Tracheotomy	4	67.2	0.03	6.10 (3.54~10.52)	<0.001	6.12 (2.06~18.18)	0.001
Central venous catheterization	3	82.7	0.003	3.22 (1.62~6.42)	0.001	2.30 (0.37~14.41)	0.37
Urinary catheter	3	0	0.89	2.14 (0.79~5.84)	0.14	2.10 (0.77~5.76)	0.15
Nasogastric tube	3	78.3	0.03	3.28 (1.85~5.83)	<0.001	3.36 (0.95~11.87)	0.06
Operation	3	0	0.75	0.78 (0.36~1.70)	0.53	0.80 (0.36~1.76)	0.57
**Antimicrobial agents**							
Carbapenems	4	23	0.27	2.82 (1.49~5.31)	0.001	2.82 (1.30~6.09)	0.008
β-lactamase inhibitor	3	85.9	0.001	7.88 (4.41~14.09)	<0.001	9.98 (1.51~65.96)	0.02
Aminoglycosides	3	27.4	0.25	4.01 (2.06~7.81)	<0.001	4.12 (1.75~9.70)	0.001
Quinolones	3	39.1	0.19	2.17 (1.21~3.89)	0.009	2.25 (1.03~4.93)	0.04
Nitroimidazoles	2	60.6	0.11	1.63 (0.43~6.24)	0.48	1.75 (0.14~22.69)	0.67

## Discussion

*Stenotrophomonas maltophilia* is widely distributed in natural environments, such as soil, water, and hospital environments, and can also parasitize the human skin, respiratory and digestive tracts. It is a very common conditional pathogen. The results of studies over the past few years have shown that the detection rate of *S. maltophilia pneumonia* is increasing year by year and has become an important pathogen of ICU infections. ICU-acquired infections associated with *S. maltophilia* are an independent risk factor for mortality in the ICU, and therefore knowledge of the risk factors for *S. maltophilia* pneumonia in the ICU and early targeted empirical treatment are key to reducing mortality from *S. maltophilia* pneumonia. In this study, we conducted a meta-analysis to screen the risk factors for *S. maltophilia* pneumonia in the ICU regarding general condition, co-morbid underlying diseases, invasive procedures, and use of antibiotics.

### Correlation of the Patient's General Condition, Co-morbid Underlying Diseases and ICU-Acquired *S. maltophilia* Pneumonia

**Figure 2:**
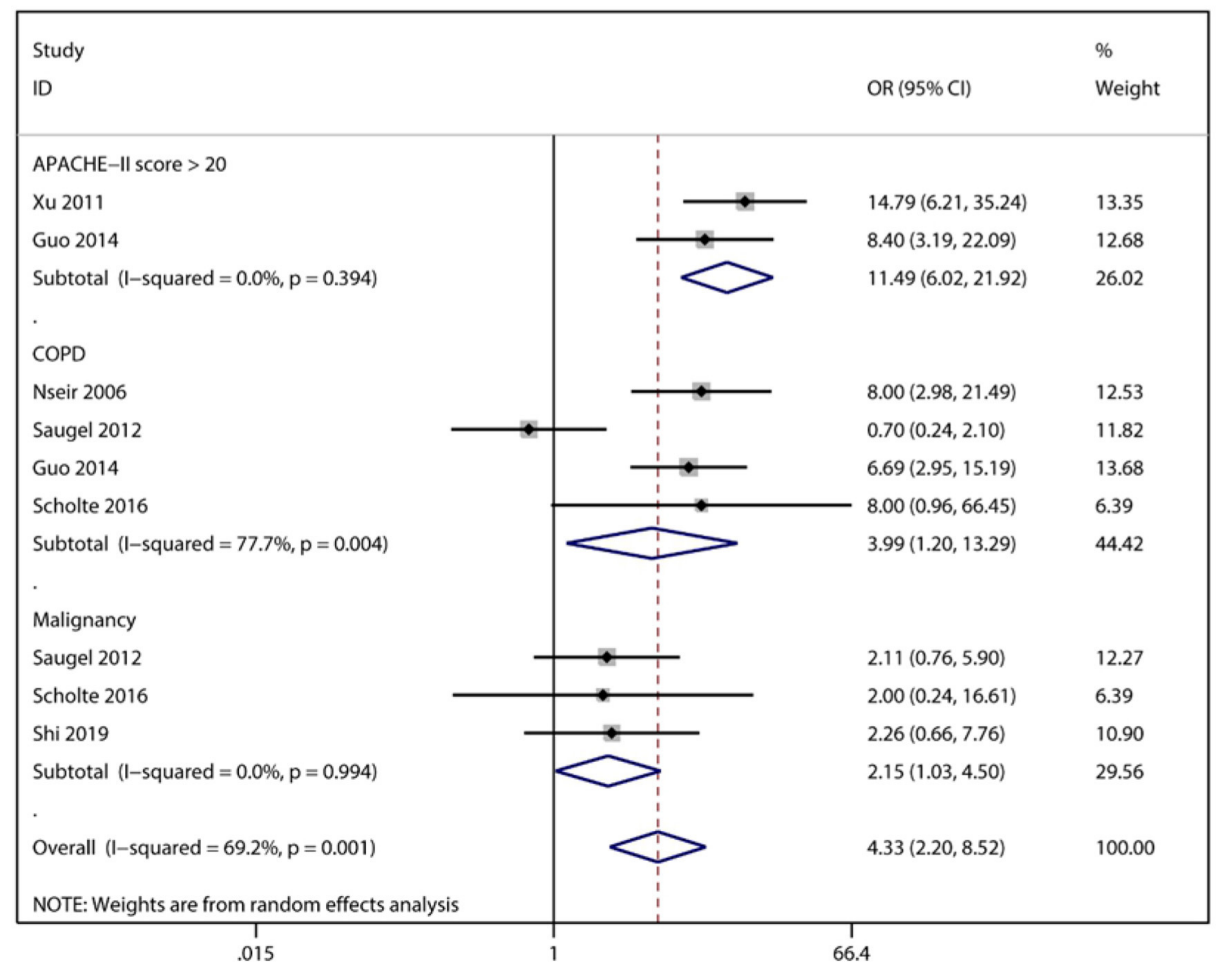
Meta-analysis results of impact of general condition and combined underlying diseases on risk factors for acquired *Stenotrophomonas maltophilia* in intensive care unit.

*S. maltophilia* pneumonia was associated with the patients' underlying disease status, and among the various underlying diseases, patients with COPD had the highest risk of infection (OR = 3.99), followed by malignant tumor (OR = 2.15). In contrast, underlying diseases such as immunodeficiency disorders, diabetes mellitus, and renal insufficiency were not associated with *S. maltophilia* pneumonia in ICU patients (Figure 2). Severity of illness is also an important factor in *S. maltophilia* pneumonia, and APACHE-II score is one of the most widely used tools for critical illness assessment (17). Furthermore, by combining the APACHE-II score of the experimental and control groups, we did not find a statistical difference between the two groups, unlike that reported by a few individual studies (9, 15). However, further analysis informed that there was a hierarchical effect between APACHE-II scores and *S. maltophilia* pneumonia, suggesting that *S. maltophilia* pneumonia is closely related to the severity of the disease. In recent years, the trend of glucocorticoid abuse in clinical practice has become more serious, and related studies have reported that long-term high-dose glucocorticoid use is a high-risk factor for multi-drug-resistant bacteria and fungal infections (18–20). However, we did not find that glucocorticoids increased the incidence of *S. maltophilia* pneumonia in the ICU, there may be bias due to different doses of glucocorticoids, course of treatment and patients' treatment response.

### Invasive Procedures and ICU-Acquired *S. maltophilia* Pneumonia

**Figure 3:**
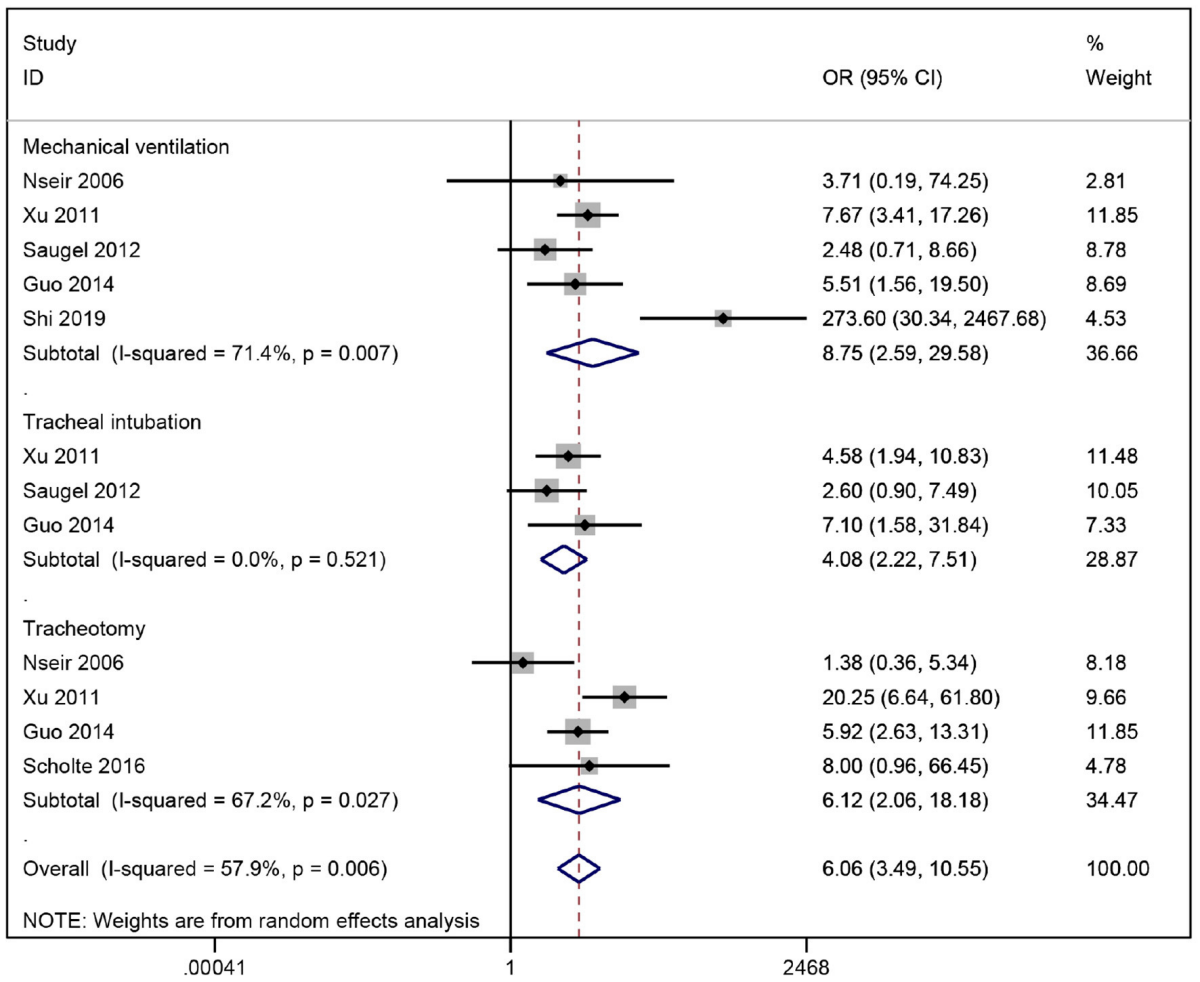
Meta-analysis results of impact of invasive operations on risk factors for acquired *Stenotrophomonas maltophilia* in intensive care unit.

Our meta-analysis indicated that among the risk factors involved in invasive maneuvers, mechanical ventilation was strongly associated with ICU-acquired *S. maltophilia* pneumonia (OR = 8.75), followed by tracheotomy (OR = 6.10) and tracheal intubation (OR = 4.25), demonstrating that invasive procedures such as mechanical ventilation, tracheal intubation and tracheotomy are high risk factors for *S. maltophilia* pneumonia in the ICU (Figure 3). Invasive procedures can breach the body's basic defense barriers. *S. maltophilia* colonized in the oral pharynx tends to form bacterial biofilms in the lining of indwelling catheters and tends to enrich at oxygen storage sites, increasing the risk of pulmonary *S. maltophilia* infection (21). In addition, the longer the duration of invasive procedures such as mechanical ventilation, the greater the risk of *S. maltophilia* infection. Guo et al. (12) have reported that the duration of invasive ventilator ventilation (>14 d) is an independent risk factor for ICU-acquired *S. maltophilia* infection. Therefore, clinical practitioners need to strictly follow the indications for invasive procedures and reduce unnecessary invasive operations, while tracheal intubation should be removed as early as possible when conditions allow to help reduce the risk of *S. maltophilia* infection. In our study, the combination of two exposure factors, central venous cannulation and indwelling nasal cannula, was more heterogeneous and less robust than other invasive procedures, and the combined results should be viewed with caution.

### The Association Between Antimicrobial Drug Use and ICU-Acquired Pneumonia With *S. maltophilia*

**Figure 4:**
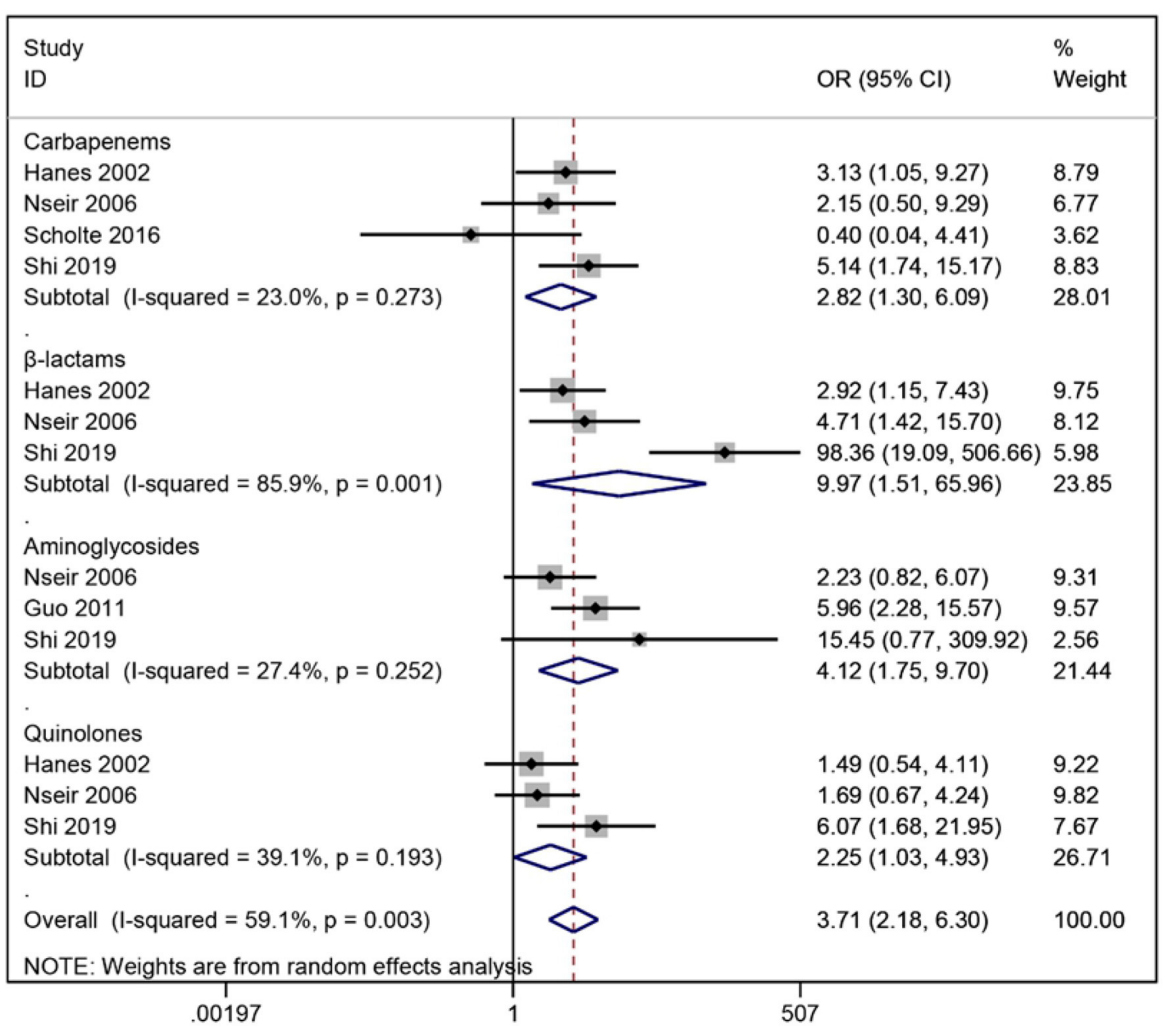
Meta-analysis results of antimicrobial drug impact of on risk factors for acquired *Stenotrophomonas maltophilia* in intensive care unit.

At present, for the treatment of antibiotic-resistant *S. maltophilia* infection, Chinese experts recommended three combined treatment modes, which are based on compound sulfamethoxazole, combined with ticarcillin clavulanate potassium, cefoperazone sulbactam, fluoroquinolone, minocycline, ceftazidime or polymyxin. Or ceftazidime-based fluoroquinolones, ticarcillin clavulanate potassium or cefoperazone sulbactam regimen; Alternatively, a polymyxin-based regimen combined with ticarcillin clavulanate potassium can be adopted (22). However, the use of antibiotics is a double-edged sword. *S. maltophilia* is naturally resistant to carbapenems, while its AAC(6')-lz acetyltransferase and pumping system make it highly resistant to aminoglycosides, and these characteristics could explain the use of both drugs to passively screen the bacterium for hospital-acquired infections due to the proliferation of dominant bacteria (23). Nseir et al. (10) found that broad-spectrum antibiotics such as fluoroquinolones and cephalosporins can also increase the rate of *S. maltophilia* pneumonia and concluded that broad-spectrum antibiotic use is more significant than carbapenems alone. This study showed that the use of β-lactamase inhibitors had the largest combined OR associated with *S. maltophilia* pneumonia (OR = 7.88), followed by aminoglycosides (OR = 4.01) and carbapenems (OR = 2.82), suggesting that the possibility of *S. maltophilia* pneumonia should be considered when clinical treatment with these three drugs is not effective (Figure 4). The long-term heavy use of broad-spectrum antibiotics is an important medical factor for hospital-acquired infections of *S. maltophilia*. Broad-spectrum antibiotics increase the risk of infection by killing other pathogens while screening out dominant species, including *S. maltophilia*. A study by Xu et al. (11) concluded that the use of ≥3 antibiotics for more than 1 week was an independent risk factor for *S. maltophilia* pneumonia in ICU patients, and therefore care should be taken to monitor the risk of infection in ICU patients using multiple antimicrobials simultaneously.

### Limitation of this Study

There are a few limitations in this study: (1) Due to the limited number of domestic and international studies on risk factors for *S. maltophilia* pneumonia in ICU and the uneven quality of the literature. Eight papers were screened strictly according to the inclusion and exclusion criteria, including five in English and three in Chinese, which may have a certain degree of publication bias. (2) Due to limited literature, some risk factor indicators in this study were not combined effectively, which may affect the study results. (3) At present, the research on related risk factors in China is not deep enough, and there is a lack of relevant prospective cohort studies. Therefore, more rigorous design, large samples, and multicenter studies are needed to clarify the risk factors for *S. maltophilia* pneumonia in the ICU.

## Conclusion

In recent years, the disease burden of hospital-acquired *S. maltophilia* pneumonia in ICU patients has been high, and resistance to the organism is increasing. *S. maltophilia* pneumonia occurs in patients with severe disease, comorbid COPD, malignancy, high APACHE-II scores, undergoing invasive procedures, and in ICU patients on broad-spectrum antibiotics due to a combination of host and medical factors. From the host side, these patients are characterized by impaired immune function, severe disease, and the need for prolonged hospitalization, which objectively contributes to the infection of conditional pathogens such as *S. maltophilia* (24). Therefore, strengthening the monitoring, prevention, and control of patients with risk factors of *S. maltophilia* infection is beneficial to reduce the risk of infection and death in ICU patients.

## Data Availability Statement

The original contributions presented in the study are included in the article/supplementary material, further inquiries can be directed to the corresponding author/s.

## Author Contributions

NW and CT: screening, data extracting, and writing. NW, CT, and LW: analysis. NW and LW: manuscript revision. LW: manuscript finalization. All authors contributed to the article and approved the submitted version.

## Conflict of Interest

The authors declare that the research was conducted in the absence of any commercial or financial relationships that could be construed as a potential conflict of interest.

## Publisher's Note

All claims expressed in this article are solely those of the authors and do not necessarily represent those of their affiliated organizations, or those of the publisher, the editors and the reviewers. Any product that may be evaluated in this article, or claim that may be made by its manufacturer, is not guaranteed or endorsed by the publisher.
